# Is the irradiated small bowel volume still a predictor for acute lower gastrointestinal toxicity during preoperative concurrent chemo-radiotherapy for rectal cancer when using intensity-modulated radiation therapy?

**DOI:** 10.1186/s13014-015-0566-6

**Published:** 2015-12-18

**Authors:** Benhua Xu, Yuyan Guo, Yuangui Chen, Haijie Lu, Tianlan Tang, Zhicao Yue, Guoxian Guan, Pan Chi, Chi Lin

**Affiliations:** Department of Radiation Oncology, The Fujian Medical University Union Hospital, Fuzhou, Fujian P.R. China 350001; Department of General Surgery, The Fujian Medical University Union Hospital, Fuzhou, Fujian P.R. China 350001; Institute of Life Sciences, Fuzhou University, Fuzhou, Fujian P.R. China 350108; Department of Radiation Oncology, University of Nebraska Medical Center, 987521 Nebraska Medical Center, Omaha, NE 68106 USA

**Keywords:** Small bowel dose volume, Acute lower gastrointestinal toxicity, Chemo-radiotherapy, Rectal cancer, Intensity-modulated radiation therapy

## Abstract

**Background:**

The small bowel (SB) represents the most important dose-limiting structure in pelvic radiotherapy (RT). However, we observed that the majority of rectal cancer patients who received preoperative pelvic intensity modulated RT (IMRT) developed acute tenesmus without watery diarrhea. The objective of this study is to determine if the RT dose to SB affects the acute lower gastrointestinal toxicity (ALGIT) in rectal cancer patients who received neoadjuvant concurrent chemotherapy-IMRT. We will also evaluate if patient and tumor factors affect the ALGIT.

**Methods:**

We retrospectively analyzed 63 rectal cancer patients that consecutively received preoperative IMRT (45 Gy for pelvis and 50 Gy for gross tumor in 25 fractions) with concurrent chemotherapy (oxaliplatin 130 mg/m^2^ on day 1 and capecitabine 825 mg/m^2^, twice per day from day 1 to day 14, week 1 and 4) between May 2012 and May 2013. The ALGIT was assessed with Common Terminology Criteria for Adverse Events version 3. The patients were stratified into two groups (with and without grade ≥2 ALGIT). The effect of SB volume receiving 5 to 40 Gy (V5 to V40) at a 5 Gy interval dose level on grade ≥2 ALGIT was evaluated. The volume of small bowel is defined as the volume of the small bowel loop. Other factors evaluated include patient’s age and gender, tumor size and location and preexisting number of daily bowel movements.

**Results:**

Overall, grade ≥2 ALGIT occurred in 57 % (36/63) patients. There was no significant difference between the two groups of patients (with and without grade ≥2 ALGIT) in SB V5 to V40, patient’s age and gender, tumor location and preexisting number of daily bowel movements. There was a significant difference between the two groups of patients in tumor volume (with grade ≥2 ALGIT: 115.5 ± 85.5 cm^3^ versus without grade ≥2 ALGIT: 58.5 ± 25.2 cm^3^, *p* = 0.0001). Multivariate analysis revealed no association between the dose SB received (V5 to V40) and the grade ≥2 ALGIT after adjusting for the tumor volume.

**Conclusions:**

With IMRT technique used in rectal cancer patients undergoing preoperative chemo-radiotherapy, the acute lower GI toxicity is not associated with small bowel V5 to V40; instead it is associated with rectal tumor size.

## Introduction

Radiation therapy plays an important role in preoperative and postoperative therapy for locally advanced rectal cancer [1–4]. Radiation-induced lower GI toxicity is the main concern. Serious acute lower GI toxicity was reported in 12 to 44 % of patients undergoing radiotherapy alone or with concurrent chemotherapy in multicenter randomized clinical trials [4, 5]. The small bowel represents the most important dose-limiting structure in the pelvic radiation therapy. Dosimetric studies revealed association between the lower GI toxicity and the irradiated small bowel volume [6, 7].

Since the German trial reported that preoperative chemo-radiotherapy improved local control and was associated with reduced toxicity as compared with postoperative chemo-radiotherapy, preoperative concurrent chemo-radiotherapy has become a standard of care for locally advanced rectal cancer (T_3–4_ and/or N positive) [4, 8]. A study has shown that the small bowel volume irradiated could be reduced in patients undergoing preoperative radiotherapy as compared with those undergoing postoperative radiotherapy [9]. Intensity-modulated radiation therapy (IMRT) has been confirmed to be advantageous at protection of the organs at risk such as bladder, femoral head and small bowel, and widely accepted as a relatively safe and effective radiotherapy method in the treatment of rectal cancer [10, 11]. Using IMRT technique further decreases the small bowel in the radiation field as compared to those using 3D conformal radiotherapy. Hence, the acute lower GI toxicity could be decreased significantly when IMRT used in preoperative chemo-radiation therapy. In clinical practice, we noticed that most rectal cancer patients who received preoperative IMRT typically presented with tenesmus and a feeling of incomplete defecation without watery diarrhea and abdominal pain. It seems that such side effects were induced rather by rectal reaction to radiation than by small bowel inflammation.

To better understand these clinical presentations, we attempted to find out if the irradiated small bowel volume still is a predictor for acute lower GI toxicity during preoperative concurrent chemo-radiotherapy for locally advanced rectal cancer when IMRT is utilized. We also examined other potential predictors for acute lower GI toxicity in this patient population.

## Patients and methods

### Patient selection

The study population consisted of 63 patients with stage II or III pathologically confirmed adenocarcinoma of the rectum who were consecutively treated with preoperative IMRT and concurrent chemotherapy between May 2012 and May 2013 in our hospital. The clinical stage and the tumor location (distance from the anal edge) were determined with physical examination and diagnostic studies, including digital rectal endoscopy, abdominal-pelvic contrast-enhanced MRI, endorectal ultrasound (EUS) and a chest CT scan. Factors recorded and analyzed include age, gender, pre-treatment number of daily bowel movements and number of daily bowel movements during the treatment (see Table 1). The study was approved by the institutional ethic committee (reference number: 2013KY011).

**Table 1:** Patient characteristics

	n (%)	Acute GI toxicity		*P* value
	*N* = 63	Grade < 2	Grade ≥2	
		*N* = 27	*N* = 36	
Age (years)				
Median (range)	55 (30–78)	55 (30–78)	57 (35–74)	
Mean (SD)	56 (10)	55 (11)	57 (10)	0.388^a^
Gender				
Male	45 (71.4)	16	29	0.092^b^
Female	18 (28.6)	11	7	
T stage				
T1	0	0	0	0.759^b^
T2	3 (4.8)	2	1	
T3	33 (52.4)	14	19	
T4	27 (42.8)	11	16	
N stage				
Negative	4 (6.3)	2	2	1.000^b^
Positive	59 (93.7)	25	34	
Tumor volume (cc)				
Median (range)	92 (20–318)	55 (20–135)	83 (26–318)	
Mean (SD)	91 (72)	59 (25)	116 (86)	0.0004^a^
Tumor location (cm from the anal verge)				
Median (range)	6 (2–12)	5 (3–10)	6(2–12)	
Mean (SD)	6 (2)	6 (2)	6 (2)	0.738^a^
Pre-existing number of daily bowel movements				
1	6 (9.5)	4	2	0.184^b^
2–3	25 (39.7)	13	12	
4–6	25 (39.7)	9	16	
≥ 7	7 (11.1)	1	6	

^a^Independent *T* test; ^b^Exact test

### Radiotherapy

All patients underwent a contrasted CT simulation with a supine position. Oral contrast was given 30 min prior to simulation in order to differentiate the small bowel from the large bowel. A planning CT scan of the lower abdomen and pelvis was obtained at 5-mm intervals from the inferior edge of the second lumbar vertebrae (L_2_) through the mid-thigh. The CT data was transferred to the Xio treatment planning system (CMS Xio Version4.6 and Monaco 3.2.0, Elekta CMS Software, Maryland Heights, MO) for delineating the target volume and organs at risk (OARs).

**Fig. 1:**
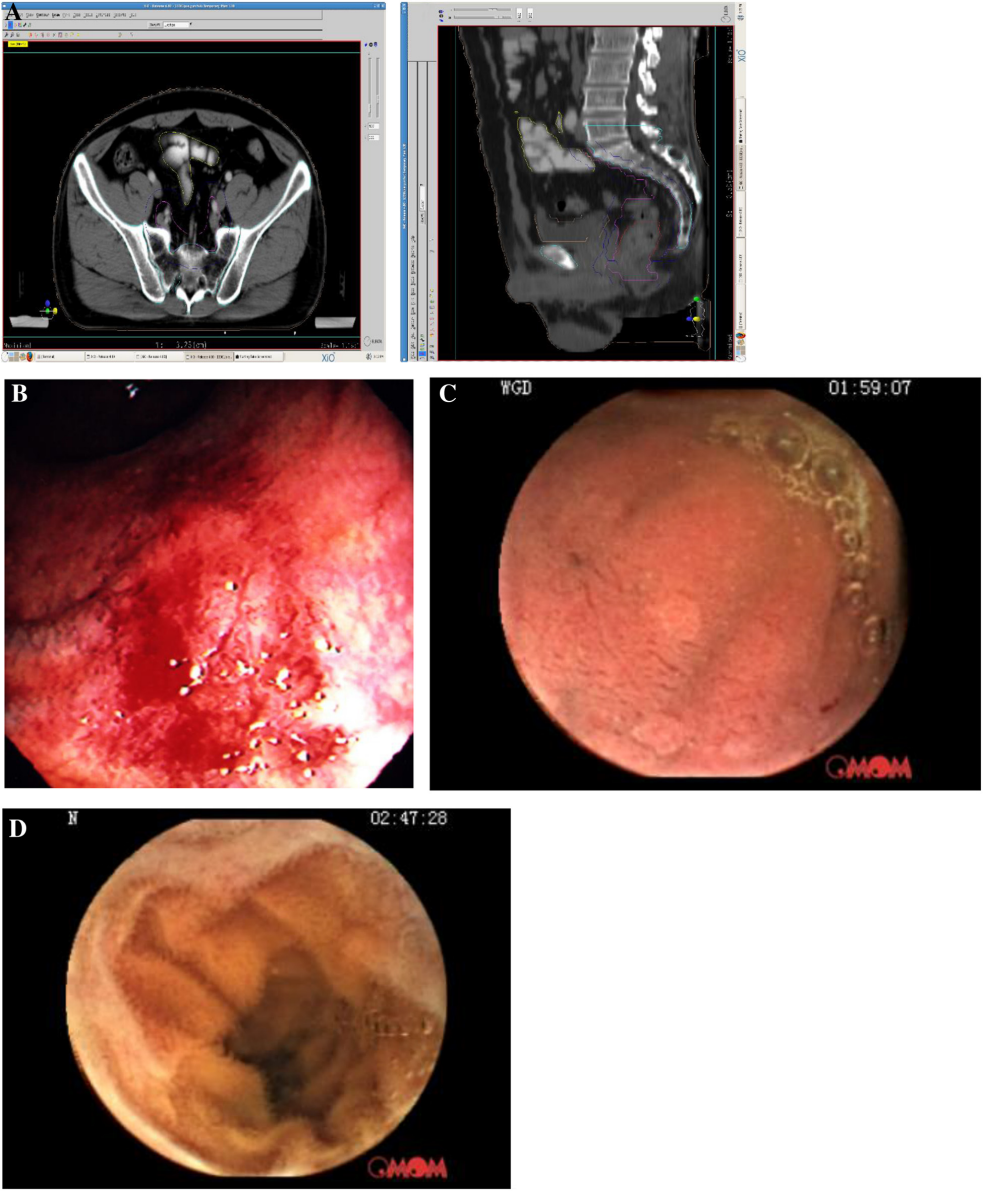
**a**. Axial (left) and sagittal (right) views of small bowel contour in a 46 year-old male patient with a stage cT3 cN+ cM0 rectal cancer treated with preoperative IMRT concurrent with chemotherapy. **b**. Reference video capsule endoscopy image for radiation induced small bowel injury: Friability and oozing blood from atrophic-appearing mucosa. (Authorization by Medscape Drugs & Diseases). **c**. Case1: A 60 year-old female patient with a stage cT_3_ cN+ cM0 rectal adenocarcinoma who presented with grade 2 tenesmus without watery stools on the day 13 of the radiotherapy which was resolved in 2 days after symptomatic treatment. Video capsule endoscopy showed normal mucosa throughout the small bowel. **d**. Case 2: A 60 year-old male patient with a stage cT_2 c_N+ cM0 rectal adenocarcinoma who did not experienced significant lower GI toxicity during the treatment course. Video capsule endoscopy showed normal mucosa throughout the small bowel

The gross tumor volume (GTV) was contoured based on clinical information, including digital rectal examination, endoscopy ultrasound and abdominopelvic MRI. The clinical target volume (CTV) included a minimum of a 3 cm craniocaudal margin to the GTV in addition to the entire mesorectum, presacral, and internal iliac lymph node drainage regions. The CTV was delineated per atlas at the RTOG web site http://www.rtog.org/Corelab/ContouringAtlases/Anorectal.aspx. Planning target volumes (PTVs) for GTV and CTV were generated with an additional 10-mm margin separately. Critical normal structures including the small bowel, bladder, femoral head, femoral neck, and pelvic bones (including sacrum, ilium, pubis and ischium) were contoured according to the pelvic normal tissue contouring guidelines of RTOG [11, 12] . The small bowel was outlined as loops containing contrast. The small bowel contouring stopped at least 2 cm above the PTV-CTV (see Fig. 1a).

The IMRT plan consisted of 5 coplanar beams with the isocenter set at the center of PTV-GTV, arranged at 55°, 115°, 180°, 245°and 310°. The dose prescribed to PTV-GTV was 50 Gy/25fractions and to PTV-CTV was 45 Gy/25fractions. The doses to OARs were limited as follows: V_45_ < 35 % for bladder, V_30_ < 15 % for femoral head, and V_30_ < 60 % and Dmax <45 Gy for small bowel. All patients were irradiated with one fraction daily for five consecutive days per week. The prescription dose was to cover at least 95 % volume of PTV.

A small bowel dose-volume histogram (DVH) was generated for each patient. The absolute and relative volumes of small bowel receiving doses between 5 Gy and 40 Gy were recorded from the DVH at 5-Gy interval (V_5_ to V_40_) according to the study of Baglan KL [6].

### Concurrent chemotherapy

The chemotherapy that patients received during the radiation therapy was per an institutional protocol [13]. All patients had two 3-week cycles of chemotherapy during the course of radiation therapy. The chemotherapy regimen included oxaliplatin (130 mg/m^2^on day-1) and capecitabine (825 mg/m^2^, twice per day from day-1 to day-14) every 3 weeks.

### Toxicity assessment

All patients received chemo-radiation therapy as inpatients. The patients were evaluated and the toxicities were recorded daily prospectively. For the purpose of this study, the daily progress notes and the final treatment summary note were reviewed to determine the lower GI toxicities during the radiation therapy course. Lower GI toxicity was assessed with Common Terminology Criteria for Adverse Events (CTCAE) version 3. Because most patients had no watery diarrhea or abdominal pain, we assessed the lower GI toxicities by using only the number of daily bowel movements [14] (see Table 2). Patients were stratified into two groups: with grade <2 and grade ≥ 2 lower GI toxicity.

**Table 2:** Scale for acute GI toxicity based on common toxicity criteria v3.0

Grade	Symptom
0	None
1	Increase of <4 stools per day over pretreatment
2	Increase of 4–6 stools per day or nocturnal stools
3	Increase of ≥7 stools per day or incontinence or need for parenteral support for dehydration
4	Physiologic consequences requiring intensive care of hemodynamic collapse

### Statistical analysis

The chi-squared test was used to compare the proportions of the two patient groups for the baseline patient characteristics and RT delivery parameters. The independent sample *T*-test was used to compare the treatment target volume, small bowel volume, the volumes of small bowel receiving doses between 5 Gy and 40 Gy between the two groups (patients with grade <2 and ≥ 2 lower GI toxicity). A *p* value of ≤ .05 was considered significant. Statistical analyses were conducted using the Statistical Analysis Systems software package, version 9.3 (SAS Institute, Cary, NC).

## Results

Overall, acute grade ≥ 2 lower GI toxicity during radiotherapy occurred in 57.1 % (36/63) patients. Among them, 14 were grade 2, 22 grade 3 and 0 grade 4. Only 4 of 36 had watery diarrhea at the second, seventh, eleventh and sixteenth fraction of radiotherapy. The remaining 27 patients experienced grade 0 and 1 lower GI toxicities. The median age of the entire group of patients was 55 (range 30–78). Most patients were male (71.4 %) and had a clinical stage of T3-4 (95.2 %) or were N positive (93.7 %). The tumor located 2–12 cm from the anal verge with a median of 6 cm. The tumor volume ranged from 20 cm^3^ to 318 cm^3^ with a median volume of 92 cm^3^ (Table 1). Patients with a larger tumor volume had a significantly higher rate of grade ≥ 2 toxicity (59 ± 25 cm^3^ vs 116 ± 86 cm^3^, *p* = 0.0004). Almost half of the patients (32/63) had pre-existing grade 2 or above frequent bowel movements secondary to rectal tumor (Table 1). It was found that 52.4 % (33/63) patients had either stable symptoms or had various extents of improvement in symptoms during the radiotherapy.

The average total contoured small bowel volume for all patients was 257 ± 152 cm^3^ and was 288 ± 193 cm^3^ and 234 ± 108 cm^3^ for patients that had lower GI toxicities of grade < 2 and ≥ 2 (*p* = 0.244), respectively. Table 3 shows that small bowel V5 to V40 did not have significant differences in patients with or without ≥ grade 2 acute lower GI toxicity.

**Table 3:** Comparison of small bowel dose-volume in patients with grade < 2 to grade ≥2 acute lower GI toxicity

Dose level	Small bowel volume(mean ± SD, cm^3^)		
(Gy)	Grade 0–1	Grade ≥2	*P* value
5	268 ± 173	217 ± 107	0.156
10	230 ± 154	189 ± 99	0.196
15	203 ± 138	169 ± 95	0.242
20	174 ± 124	143 ± 86	0.247
25	127 ± 105	103 ± 68	0.272
30	75 ± 78	60 ± 49	0.367
35	47 ± 63	37 ± 37	0.445
40	30 ± 49	23 ± 24	0.429

Factors such as age, gender, T stage, N stage, tumor volume, tumor location, pre-existing number of daily bowel movement and small bowel V5-V40 were analyzed in the univariate logistic regression model (Table 4). All but tumor volume (*p* = 0.002), pre-existing number of daily bowel movements (*p* = 0.06) and gender (*p* = 0.07) were not significantly correlated with grade ≥ 2 acute lower GI toxicity. Therefore, only tumor volume, pre-existing number of daily bowel movements and gender were included in the multivariate logistic regression analysis. Multivariate analysis (Table 4) revealed that tumor volume (OR 0.147, 95 % CI 0.043–0.499) and pre-existing number of daily bowel movements (OR 0.272, 95 % CI 0.080–0.922) but not gender were significantly correlated with grade ≥ 2 acute lower GI toxicity. Forcing V5-V40 into the model showed that small bowel V5-V40 was not significantly correlated with grade ≥ 2 acute lower GI toxicity.

**Table 4:** Factors associated with grade ≥ 2 lower GI toxicity

Factors	Median	Univariate		Multivariate	
		OR (95 % CL)	*P* value	OR (95 % CL)	*P* value
Age (years)	≤55/>55	0.716 (0.259–1.946)	0.513		
Gender	Female/Male	0.351 (0.114–1.084)	0.069	0.376 0.108 1.308	0.124
T stage	≤ T3/>T3	0.859 (0.313–2.361)	0.769		
N stage	N-/N+	0.735 (0.097–5.581)	0.766		
Tumor volume (cc)	≤64/> 64	0.185 (0.062–0.550)	0.002	0.147 0.043 0.499	0.002
Tumor location^a^	≤6/>6	0.700 (0.248–1.978)	0.501		
Pre-existing BM^b^	<4/≥ 4	0.374 (0.134–1.048)	0.061	0.272 0.080 0.922	0.036
Small bowel V5 (cc)	≤213/>213	1.562 (0.572–4.265)	0.384		
Small bowel V10 (cc)	≤191/>191	1.562 (0.572–4.265)	0.384		
Small bowel V15 (cc)	≤174/>174	1.818 (0.662–4.995)	0.246		
Small bowel V20 (cc)	≤146/>146	1.818 (0.662–4.995)	0.246		
Small bowel V25 (cc)	≤100/>100	1.626 (0.593–4.458)	0.345		
Small bowel V30 (cc)	≤53/>53	1.818 (0.662–4.995)	0.246		
Small bowel V35 (cc)	≤23/>23	0.550 (0.200–1.511)	0.246		
Small bowel V40 (cc)	≤11/>11	0.640 (0.234–1.747)	0.384		

^a^Distance from the anal verge; ^b^pre-existing number of daily bowel movement

## Discussion

The most interesting finding of this study is no correlations between the dose-volume parameters of small bowel (V_5_ to V_40_) and the acute lower GI toxicity during preoperative concurrent chemo-radiotherapy for rectal cancer when IMRT is used. This study also revealed that the rectal tumor volume is a predictor of grade ≥ 2 lower GI toxicity. To our knowledge, this is the first study to report that with IMRT, the acute lower GI toxicity in this patient population is mainly influenced by rectal tumor volume but not small bowel dose-volume parameters.

### Preoperative radiotherapy with IMRT decreased small bowel volume in the pelvic radiation field

Minsky et al. showed a dramatic decrease of small bowel volume in the radiation field for patients that received preoperative radiotherapy when compared with postoperative radiotherapy (212 ± 44 cm^3^ versus 462 ± 129 cm^3^, p = 0.002) [9]. Sauer et al. found that ≥ grade 3 diarrhea occurred in patients treated preoperatively significantly less frequent than those treated postoperatively (12 % vs. 18 %, *p* = 0.04) [4]. To further reduce small bowel volume in the radiation field, IMRT has been used in several studies in the preoperative chemo-radiation therapy for rectal cancer [15, 16]. Two dosimetric studies showed that the small bowel volume was reduced significantly in the radiation field with IMRT [17, 18]. In the study by Arbea L et al., the small bowel V_40_ with IMRT was only one third of the V_40_ treated with conventional 3-field technique (68.9 cc vs. 178.3 cc, *p* < 0.01) [19]. In our study, all patients received preoperative radiotherapy with IMRT technique. The small bowel V_40_ in the current study was only 23–30 cc which is smaller than the small bowl V40 (68.9 cc) in patients treated with IMRT in the study of Arbea et al. Our study suggests that the small bowel V_5_-V_20_ are no longer predictors for grade ≥ 2 lower GI toxicity when an irradiated small bowl volume is reduced to a very low level by using modern radiotherapy technique such as IMRT.

### Lower GI toxicity is the consequence of rectal irradiation

It is well known that the symptoms of small bowel and rectal injury are different. The symptoms from acute small bowel injury include watery diarrhea, colicky abdominal pain, bloating, loss of appetite, nausea and dehydration [20]. On the other hand, the symptoms from rectal injury typically include soft or diarrhea-like stools, rectal pain and tenesmus (a sense of rectal distention with cramping and frequency) [21]. Studies have shown that patients treated with chemotherapy alone had a risk of around 20 % in developing ≥ grade 3 treatment-related diarrhea [22] and adding chemotherapy concurrently to radiotherapy increased the frequency and severity of diarrhea [23]. In theory, diarrhea caused by chemotherapy should occur in the course of chemotherapy and could be cured in a short duration. In our study, most patients presented tenesmus. We believe that the occasional watery diarrhea of 4 patients in our study was caused by concurrent chemotherapy since they only occurred during the week of receiving chemotherapy and was cured in a week.

### Primary tumor volume influences the lower GI toxicity

Another finding in this study was that the most common acute lower GI toxicity was tenesmus. The gross tumor volume was the only independent influencing factor for the acute lower GI toxicity. The larger the tumor volume the more frequent bowel movements (115.5 ± 85.5 cm^3^ versus 58.5 ± 25.2 cm^3^ for grade ≥ 2 and grade 0–1, *p* = 0.000). Given that most patients had no watery diarrhea, we believe that the named lower GI acute toxicity is a rectal reaction to the tumor and to the radiation injury. In the study of Myerson RJ et al., although they used the 3D-CRT technique, they also found that proctitis was the most common acute toxicity (5/37) during preoperative chemo-radiotherapy and associated with large tumors (PTV ≥ 500 cc). There was only one patient who had enteritis during the treatment course [24].

### Wireless whole gastrointestinal video capsule endoscopy

The acute toxicity of the small bowel is to a large extent a result of clonogenic and apoptotic cell death in the crypt epithelium, resulting in insufficient replacement of the villus epithelium, breakdown of the mucosal barrier, mucositis, and prominent compensatory and proliferative reactions (see Fig. 1b) [25]. The best way to observe the small bowl reaction to radiation is to use the wireless whole gastrointestinal video capsule endoscopy (VCE). Video capsule endoscopy enables excellent visualization of the small bowel mucosa [26]. Only a pilot study and a case report demonstrated the usefulness of VCE for radiation-induced late small bowel injury [27, 28]. We elected 2 patients, one had grade 2 and the other had grade 0 lower GI toxicity, to undergo VCE (Model of JS-ME-II, Chongqing Jinshan Science and Technolgy (group) Co.,Ltd) examination in 2 to 3 days after the completion of preoperative chemo-radiotherapy in order to observe the changes in the small bowel mucosa. The duration of both VCE examinations were around 9 h and both showed normal small bowel mucosa (see Fig. 1c and d). The small bowel V_5_-V_40_ of these two patients are shown in Table 5. These findings directly confirmed our results that clinical lower GI toxicity in pelvic IMRT is not associated with the dose-volume parameters of small bowel.

**Table 5:** Small bowel dose-volume for the two patients who underwent VCE examination

Dose level	Small bowel volume (cm^3^)	
(Gy)	Case 1	Case 2
5	330	183
10	295	159
15	281	142
20	254	116
25	192	90
30	108	44
35	64	18
40	40	9

### Quantifying the lower GI toxicity

Although there were many reports concerning the acute and chronic small bowel toxicity in pelvic radiation therapy, little data has been published quantifying the details of the toxicity such as extent, timing, duration, correlation with chemotherapy, etc. It is very hard to evaluate the lower GI toxicity via the status of stools for rectal cancer, because for most patients, the most common symptom at the time of diagnosis is increasing frequency of stools with or without mixed blood. Among the 63 patients in our study, the number of pre-existing daily bowel movements was found to be 1 time in 6 (9.5 %) patients, 2–3 times in 25 (39.7 %) patient, 4–6 times in 25(39.7 %) patients and ≥ 7 stools in 7 patients (11.1 %) (see Table 1). Almost half of these patients presented with grade ≥ 2 bowel toxicity before initiating radiotherapy treatment. Moreover, both RTOG [29] and CTCAE [14] toxicity scales for lower GI toxicity are simple and rapidly assessable, but provide limited information. For example, they do not assess the development of anorectal symptoms such as tenesmus which is the most common symptom during the pelvic irradiation, especially for rectal cancer treated with IMRT technique. A modified questionnaire from the Inflammatory Bowel Disease Questionnaire (IBDQ) and Vaizey Incontinence questionnaire may be more sensitive and useful for toxicity data collection during the treatment and follow-up [30, 31]. Such questionnaires include much more information when compared to RTOG and CTCAE scales.

Limitations of this study include its retrospective nature, a lack of information on total small bowel volume and details of toxicity.

## Conclusion

When small bowel volume in the radiation field was reduced to a low level with IMRT, it was no longer as an influencing factor for acute lower GI toxicity. The radiation-induced lower GI symptoms such as tenesmus without watery diarrhea during pelvic IMRT may be caused by the rectal tumor itself and rectal reaction to radiotherapy. A future prospective study is warranted to investigate the details of lower GI toxicity during treatment and early follow-up, by using a real-time recorded clinical note, modified questionnaire and whole gastrointestinal wireless capsule endoscopy.

## References

[1] Prolongation of the disease-free interval in surgically treated rectal carcinoma. Gastrointestinal Tumor Study Group. The New England journal of medicine.N Engl J Med. 1985, 312(23):1465–1472. 10.1056/NEJM198506063122301 2859523

[2] O’Connell MJ, Martenson JA, Wieand HS, Krook JE, Macdonald JS, Haller DG, Mayer RJ, Gunderson LL, Rich TA (1994). Improving adjuvant therapy for rectal cancer by combining protracted-infusion fluorouracil with radiation therapy after curative surgery. N Engl J Med.

[3] Pasetto LM, Pucciarelli S, Agostini M, Rossi E, Monfardini S (2004). Neoadjuvant treatment for locally advanced rectal carcinoma. Crit Rev Oncol Hematol.

[4] Sauer R, Becker H, Hohenberger W, Rodel C, Wittekind C, Fietkau R, Martus P, Tschmelitsch J, Hager E, Hess CF (2004). Preoperative versus postoperative chemoradiotherapy for rectal cancer. N Engl J Med.

[5] Roh MS, Colangelo LH, O’Connell MJ, Yothers G, Deutsch M, Allegra CJ, Kahlenberg MS, Baez-Diaz L, Ursiny CS, Petrelli NJ (2009). Preoperative multimodality therapy improves disease-free survival in patients with carcinoma of the rectum: NSABP R-03. J Clin Oncol.

[6] Baglan KL, Frazier RC, Yan D, Huang RR, Martinez AA, Robertson JM (2002). The dose-volume relationship of acute small bowel toxicity from concurrent 5-FU-based chemotherapy and radiation therapy for rectal cancer. Int J Radiat Oncol Biol Phys.

[7] Banerjee R, Chakraborty S, Nygren I, Sinha R (2013). Small bowel dose parameters predicting grade >/= 3 acute toxicity in rectal cancer patients treated with neoadjuvant chemoradiation: an independent validation study comparing peritoneal space versus small bowel loop contouring techniques. Int J Radiat Oncol Biol Phys.

[8] Bosset JF, Collette L, Calais G, Mineur L, Maingon P, Radosevic-Jelic L, Daban A, Bardet E, Beny A, Ollier JC (2006). Chemotherapy with preoperative radiotherapy in rectal cancer. N Engl J Med.

[9] Minsky BD, Conti JA, Huang Y, Knopf K (1995). Relationship of acute gastrointestinal toxicity and the volume of irradiated small bowel in patients receiving combined modality therapy for rectal cancer. J Clin Oncol.

[10] Mok H, Crane CH, Palmer MB, Briere TM, Beddar S, Delclos ME, Krishnan S, Das P (2011). Intensity modulated radiation therapy (IMRT): differences in target volumes and improvement in clinically relevant doses to small bowel in rectal carcinoma. Radiat Oncol.

[11] Samuelian JM, Callister MD, Ashman JB, Young-Fadok TM, Borad MJ, Gunderson LL (2012). Reduced acute bowel toxicity in patients treated with intensity-modulated radiotherapy for rectal cancer. Int J Radiat Oncol Biol Phys.

[12] Gay HA, Barthold HJ, O’Meara E, Bosch WR, El Naqa I, Al-Lozi R, Rosenthal SA, Lawton C, Lee WR, Sandler H (2012). Pelvic normal tissue contouring guidelines for radiation therapy: a Radiation Therapy Oncology Group consensus panel atlas. Int J Radiat Oncol Biol Phys.

[13] Xu BH, Chi P, Guo JH, Guan GX, Tang TL, Yang YH, Chen MQ, Song JY, Feng CY (2014). Pilot study of intense neoadjuvant chemoradiotherapy for locally advanced rectal cancer: retrospective review of a phase II study. Tumori.

[14] Trotti A, Byhardt R, Stetz J, Gwede C, Corn B, Fu K, Gunderson L, McCormick B, Morrisintegral M, Rich T (2000). Common toxicity criteria: version 2.0. an improved reference for grading the acute effects of cancer treatment: impact on radiotherapy. Int J Radiat Oncol Biol Phys.

[15] Callister MD, Ezzell GA, Gunderson LL (2006). IMRT Reduces the Dose to Small Bowel and Other Pelvic Organs in the Preoperative Treatment of Rectal Cancer. Int J Radiat Oncol Biol Phys.

[16] Engels B, De Ridder M, Tournel K, Sermeus A, De Coninck P, Verellen D, Storme GA (2009). Preoperative helical tomotherapy and megavoltage computed tomography for rectal cancer: impact on the irradiated volume of small bowel. Int J Radiat Oncol Biol Phys.

[17] Duthoy W, De Gersem W, Vergote K, Boterberg T, Derie C, Smeets P, De Wagter C, De Neve W (2004). Clinical implementation of intensity-modulated arc therapy (IMAT) for rectal cancer. Int J Radiat Oncol Biol Phys.

[18] Guerrero Urbano MT, Henrys AJ, Adams EJ, Norman AR, Bedford JL, Harrington KJ, Nutting CM, Dearnaley DP, Tait DM (2006). Intensity-modulated radiotherapy in patients with locally advanced rectal cancer reduces volume of bowel treated to high dose levels. Int J Radiat Oncol Biol Phys.

[19] Arbea L, Ramos LI, Martinez-Monge R, Moreno M, Aristu J (2010). Intensity-modulated radiation therapy (IMRT) vs. 3D conformal radiotherapy (3DCRT) in locally advanced rectal cancer (LARC): dosimetric comparison and clinical implications. Radiat Oncol.

[20] Stacey R, Green JT (2014). Radiation-induced small bowel disease: latest developments and clinical guidance. Ther Advanc Prev Chronic Dis.

[21] Michalski JM, Gay H, Jackson A, Tucker SL, Deasy JO (2010). Radiation dose-volume effects in radiation-induced rectal injury. Int J Radiat Oncol Biol Phys.

[22] Cassidy J, Tabernero J, Twelves C, Brunet R, Butts C, Conroy T, Debraud F, Figer A, Grossmann J, Sawada N (2004). XELOX (capecitabine plus oxaliplatin): active first-line therapy for patients with metastatic colorectal cancer. J Clin Oncol.

[23] Miller RC, Martenson JA, Sargent DJ, Kahn MJ, Krook JE (1998). Acute treatment-related diarrhea during postoperative adjuvant therapy for high-risk rectal carcinoma. Int J Radiat Oncol Biol Phys.

[24] Myerson RJ, Valentini V, Birnbaum EH, Cellini N, Coco C, Fleshman JW, Gambacorta MA, Genovesi D, Kodner IJ, Picus J (2001). A phase I/II trial of three-dimensionally planned concurrent boost radiotherapy and protracted venous infusion of 5-FU chemotherapy for locally advanced rectal carcinoma. Int J Radiat Oncol Biol Phys.

[25] Hauer-Jensen M, Wang J, Denham JW (2003). Bowel injury: current and evolving management strategies. Semin Radiat Oncol.

[26] Kopylov U, Nemeth A, Koulaouzidis A, Makins R, Wild G, Afif W, Bitton A, Johansson GW, Bessissow T, Eliakim R (2015). Small bowel capsule endoscopy in the management of established Crohn’s disease: clinical impact, safety, and correlation with inflammatory biomarkers. Inflamm Bowel Dis.

[27] Kim HM, Kim YJ, Kim HJ, Park SW, Bang S, Song SY (2011). A pilot study of capsule endoscopy for the diagnosis of radiation enteritis. Hepato-Gastroenterology.

[28] Lee DW, Poon AO, Chan AC (2004). Diagnosis of small bowel radiation enteritis by capsule endoscopy. Hong Kong Med J.

[29] Cox JD, Stetz J, Pajak TF (1995). Toxicity criteria of the Radiation Therapy Oncology Group (RTOG) and the European Organization for Research and Treatment of Cancer (EORTC). Int J Radiat Oncol Biol Phys.

[30] Khalid U, McGough C, Hackett C, Blake P, Harrington KJ, Khoo VS, Tait D, Norman AR, Andreyev HJ (2006). A modified inflammatory bowel disease questionnaire and the Vaizey Incontinence questionnaire are more sensitive measures of acute gastrointestinal toxicity during pelvic radiotherapy than RTOG grading. Int J Radiat Oncol Biol Phys.

[31] Olopade FA, Norman A, Blake P, Dearnaley DP, Harrington KJ, Khoo V, Tait D, Hackett C, Andreyev HJ (2005). A modified Inflammatory Bowel Disease questionnaire and the Vaizey Incontinence questionnaire are simple ways to identify patients with significant gastrointestinal symptoms after pelvic radiotherapy. Br J Cancer.

